# Distinguishing Isomeric Caffeine Metabolites through Protomers and Tautomers Using Cryogenic Gas-Phase Infrared Spectroscopy

**DOI:** 10.1021/acs.analchem.5c05164

**Published:** 2025-09-25

**Authors:** Niklas Geue, Gurpur Rakesh D. Prabhu, Eleonora Renzi, Caitlin Walton-Doyle, Gerard Meijer, Gert von Helden, Kevin Pagel

**Affiliations:** † Institute of Chemistry and Biochemistry, 28259 Freie Universität Berlin, Altensteinstraße 23a, Berlin 14195, Germany; ‡ Department of Molecular Physics, Fritz-Haber-Institut der Max-Planck-Gesellschaft, Faradayweg 4−6, Berlin 14195, Germany

## Abstract

Caffeine is metabolized through various pathways in the human body, with the primary two steps yielding isomeric products. Distinguishing these metabolites is crucial for mass spectrometry-based metabolomics, for example, to assess specific drug interactions. Here, we investigate the gas-phase structures of caffeine and its metabolites—theophylline, theobromine, paraxanthine, 1-methylxanthine, 3-methylxanthine, and 7-methylxanthine—in their respective protonated ions using cryogenic gas-phase infrared spectroscopy, supported by density functional theory. The analytes exhibit varying preferences for protonation and tautomerism, particularly N9 protonation and, where applicable, a tendency for N3O2 and N1O2 amide–imidic acid and N7N9 imine–imine tautomerism. We further demonstrate that the two isomeric sets of caffeine metabolites can easily be distinguished with gas-phase IR spectroscopy, paving the way for robust identification of such molecules in metabolomics using hyphenated gas-phase techniques.

## Graphical abstract

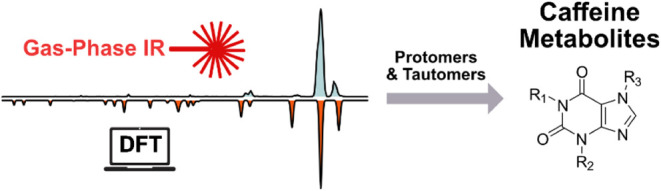

## Introduction

Caffeine is the most widely consumed psychoactive substance worldwide, found in coffee, tea, chocolate, and various pharmaceutical products.^1^ Upon ingestion, it undergoes hepatic metabolism primarily via cytochrome P450 enzymes to produce isomeric dimethylxanthines (theobromine, theophylline, and predominantly paraxanthine) and subsequently methylxanthines (1-, 3-, and 7-methylxanthine).^2^ These metabolites possess distinct physiological activities and pharmacokinetic profiles, making accurate identification and quantification critical for understanding individual metabolic variation, guiding therapeutic monitoring, and supporting toxicological studies.^2^

The accurate structural characterization of isomeric metabolites is challenging, and orthogonal mass spectrometry-based techniques, such as ion mobility mass spectrometry (IM-MS), are emerging as robust and high-throughput tools for metabolomics.^3–6^ Ion mobility (IM) separates ions by their size and shape, which is commonly achieved by measuring the time ions need to traverse a gas-filled cell. Larger and more extended ions undergo more collisions with the gas, leading to higher so-called arrival times, whereas smaller and more compact analytes collide less often with the buffer gas, resulting in lower arrival times.^7–10^ While IM can, in principle, separate isomers, it can be limited by insufficient resolution and the inability to directly identify the analyte of interest.^10,11^

The IM-MS analysis of caffeine and its metabolites is complicated by the occurrence of different protonation sites (protomers) and types of tautomerism (tautomers, Figure 1), as demonstrated in a recent study by Sepman et al.^12^ The authors used cyclic IM-MS^13^ to characterize different protomers and tautomers of caffeine metabolites, showing that, for most of the metabolites, two distinct species could be separated. However, at the resolution achieved with this instrument, their arrival times are often similar to those of the protomers and tautomers corresponding to the isomeric metabolites.

**Figure 1:**
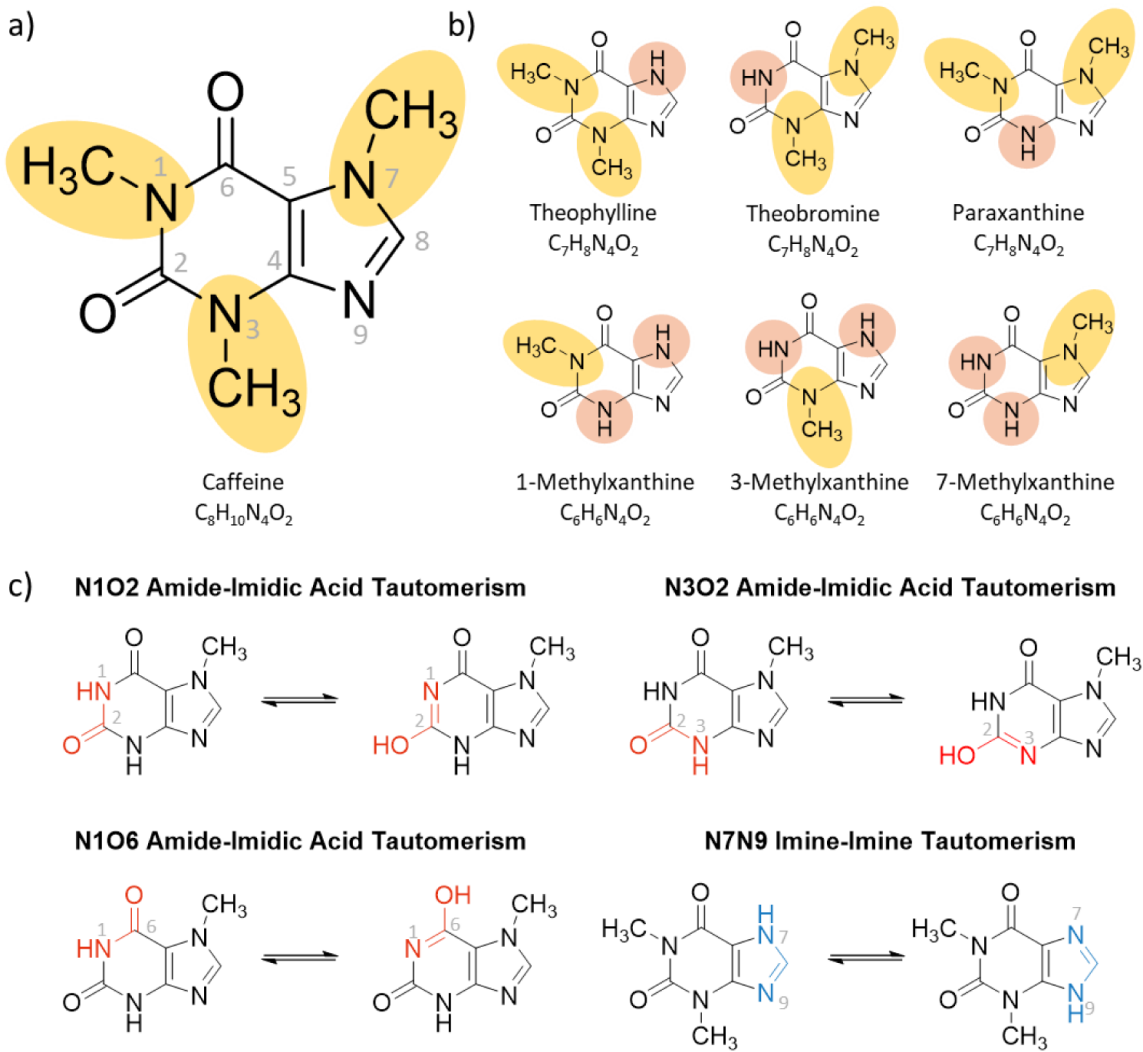
Structures and masses of a) caffeine (including atom labeling) and b) the primary isomeric metabolites (theophylline, theobromine, and paraxanthine), and secondary isomeric metabolites (1-methylxanthine, 3-methylxanthine, 7-methylxanthine). c) Schematics for different forms of amide–imidic acid tautomerism (N1O2, N1O6, N3O2; red) and imine–imine tautomerism (N7N9; blue).

Sepman et al. further tried to distinguish the isomeric metabolites via the fragmentation of the mobility-separated protomers and tautomers of each metabolite.^12^ Even when this additional separation dimension was exploited, prior to MS^2^, not all isomeric metabolites could be distinguished. Additionally, their separation and identification with LC-MS methods have traditionally been challenging.^14,15^ When separation was achieved, it required extensive method optimization, and some transitions remained identical across isomers. During method development, analytes frequently coeluted, demonstrating the inherent difficulty of resolving these metabolites with LC-MS/MS.^16^

Gas-phase infrared (IR) spectroscopy is a powerful tool to probe the structure of gaseous ions, enabling the differentiation of closely related molecular species with high specificity. This is realized by irradiating ions with photons at their resonant frequencies, which leads to vibrational excitation commonly followed by fragmentation (in infrared multiple photon dissociation, IRMPD).^17,18^ The intensity of the fragment ions as a function of wavelength yields an IR spectrum, which, in turn, is compared to those computed from vibrational frequencies based on density functional theory (DFT) calculations.^19^ Other gas-phase IR spectroscopy approaches include messenger-tagging spectroscopy, where ions are tagged with gases and untagged upon irradiation, or gas-phase IR spectroscopy with helium nanodroplets. In the latter, ions are encapsulated in helium droplets, which evaporate when they absorb energy from resonant photons, releasing the ions and enabling their detection.^19^ This technique results in less spectral congestion and higher resolution compared to IRMPD, and was therefore used in this study.

Gas-phase IR spectroscopy has shown great promise for metabolite identification.^20–22^ For example, Houthuijs et al. recently introduced an *in silico* infrared spectral library for 4500 metabolites, greatly facilitating identification when using gas-phase IR spectroscopy in metabolomics workflows.^22^ For the analysis of complex mixtures, the combination of IM-MS with gas-phase IR spectroscopy has shown strong prospects for robust identification and potential in quantification.^23–25^ Establishing workflows that enable the separation and identification of metabolites with these techniques from complex mixtures is therefore highly relevant.^26^

In this study, we apply gas-phase IR spectroscopy, supported by DFT calculations, to characterize and distinguish the protomers of caffeine and its regioisomeric metabolites: theophylline, theobromine, and paraxanthine, as well as 1-methylxanthine, 3-methylxanthine, and 7-methylxanthine (Figure 1). By characterizing and comparing experimental IR spectra with theoretical models of different tautomers and protomers, we demonstrate the utility of this approach for resolving isomeric complexity within a key biochemical pathway, offering broader implications for structural metabolomics.

## Methods

Caffeine and its metabolites were obtained from TCI Chemicals and abcr GmbH, and solutions of 4:1 acetonitrile:water (v/v) were prepared with 0.5% formic acid. Final concentrations of 200 μM (caffeine, theobromine, theophylline, paraxanthine) and 1 mM (1-methylxanthine, 3-methylxanthine, 7-methylxanthine) were used, respectively. Samples were transferred to the gas phase using nanoelectrospray ionization (nESI) from capillaries with an inner tip diameter of 1–2 μm, which were home-pulled glass on a pipette puller (Model: *p*-2000; Sutter Instrument Company). A capillary voltage of 0.8–1.0 kV was applied through a Pt wire.

The instrumentation used for helium nanodroplet gas-phase IR spectroscopy has been described in the literature.^27,28^ A quadrupole performs the *m*/*z*-selection of the protonated ions of interest. The ions are subsequently transferred to a hexapole ion trap, where they are cooled to ca. 90 K by collisions with helium gas, which is in turn cooled by liquid nitrogen. Superfluid helium nanodroplets (*T* = 0.37 K) are generated by a pulsed Even–Lavie valve^29^ and pick up the trapped ions, leading them to the detection region. The beam of doped droplets interacts with an IR beam generated by the tunable Fritz Haber Institute free-electron laser (FHI-FEL).^30^ The excitation of the protonated ions inside the helium droplets with resonant IR photons (*ṽ* = 1000–1850 cm^–1^) leads to the release of the ions, which are then detected with a time-of-flight mass analyzer. An IR spectrum is obtained by plotting the ion count against the wavenumber. Due to the nature of the multiphoton absorption process, the intensities in the obtained IR spectrum do not scale linearly across different wavenumbers. As a correction, the ion signal is divided by the energy of the IR macropulse.

Experimental IR spectra are compared to those obtained from DFT calculations. For each compound, reasonable candidate protomers and tautomers were generated and optimized at the PBE0+D3/6–311+G­(d,p)^31,32^ level of theory in Gaussian 16.^33^ Harmonic frequencies were computed, and all calculated IR spectra were normalized and scaled by an empirical factor of 0.965.^34^ The basis set and functional were selected based on prior experience when comparing simulated IR spectra to those obtained experimentally from our home-built helium nanodroplet instrument.^28,35,36^ The free energy at 90 K was used to rank the reoptimized protomers and tautomers. Although sufficient for the purpose of this manuscript, the precision of DFT optimizations could be further improved by incorporating conformational searches to elucidate the global energy minimum of each protomer/tautomer. This is not critical due to the rigidity of the analytes; however, it could result in, for example, a different orientation of protons within the molecule, leading to slightly different energetics.

## Results

The protonated cations of caffeine and its six metabolites were structurally characterized by cryogenic IR spectroscopy and DFT calculations. For this purpose, each precursor ion was transferred to the gas phase via nanoelectrospray ionization, yielding the protonated cations as the main peaks (Figures S1–S7). The experimental IR spectra mainly show two regions: the fingerprint region (1000–1500 cm^–1^) and the functional group region (1500–1850 cm^–1^). The former contains mainly the C–O, C–C, and C–N stretching as well as C–H bending vibrations. It can be challenging to model this region due to the complexity of C–C and C–O stretching vibrations.^34^ The second region, the functional group region, is largely populated by C=O, C=N, and C=C stretching vibrations and is preferred for diagnostic assignments. Structural assignments of the protomers were achieved by comparing the experimental spectra to those based on harmonic frequencies of DFT-optimized candidate structures (Figures S8–S14 for DFT-optimized structures, Tables S1–S7 for ion energetics). This included protonation at heteroatoms (O2, O6, and N9) as well as their tautomers following amide–imidic acid tautomerism (proton transfer between N1 and O2; N1 and O6; as well as N3 and O2) and/or imine–imine tautomerism (proton transfer between N7 and N9; Figure 1a for atom labeling and Figure 1c for tautomerism).

We first aimed to verify the structure of protonated caffeine, which had previously been reported to form a single isomer^12,37^ with the proton located at N9.^38^ This ion allowed us to establish the most suitable macropulse energy of the free-electron laser. The maximum macropulse energy (60 mJ) and the attenuation to one-third of this value (20 mJ, Figure 2) were tested. As expected, the IR spectrum at maximum macropulse energy shows more bands with high intensity; however, the signals are broad and appear saturated. At 20 mJ, fewer bands occur, but with narrow peak shapes and different intensities per band, indicative of an undersaturated spectrum. The strongest experimental bands in the functional group region were assigned as C=O stretching (C2=O: 1771 cm^–1^, C6=O: 1742 cm^–1^) and C4=N3 stretching (1687 cm^–1^). While both spectra clearly confirm the assignment of the Pro_N9 isomer of caffeine, displayed in Figure 2, we chose to go forward with 20 mJ macropulse energy for the measurement of the six metabolites since the resolution in the diagnostic functional group region was higher.

**Figure 2:**
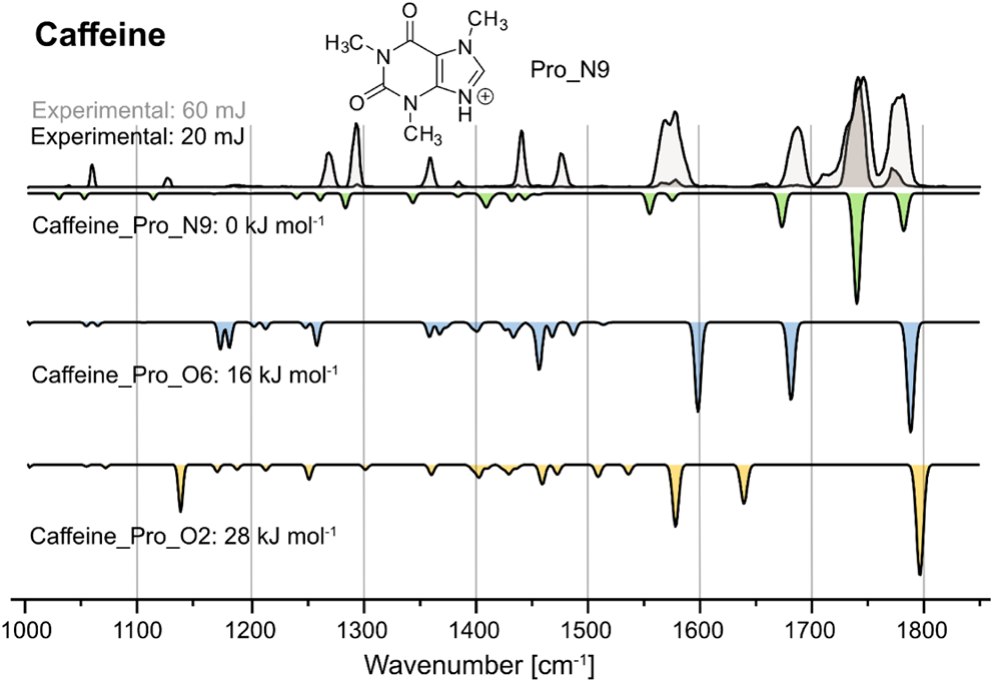
Cryogenic infrared spectra of the protonated cation of caffeine at 20 mJ (front) and 60 mJ (back) FEL macropulse energies. Computed IR spectra are shown as inverted traces for structures with N9-protonation (green), O6-protonation (blue), and O2-protonation (yellow). Both experimental traces can be assigned to Caffeine_Pro_N9, for which the structure is shown.

Next, we measured the IR spectra of the three primary caffeine metabolites: theophylline, theobromine, and paraxanthine (Figure 3a–c), in which the methyl groups at N7, N1, and N3 are substituted by protons, respectively (Figure 1b). This substitution can lead to the occurrence of N7N9 imine–imine tautomerism for theophylline, as well as amide–imidic acid tautomerism via N3O2 for paraxanthine or via N1O2 or N1O6 for theobromine (Figure 1c).

**Figure 3:**
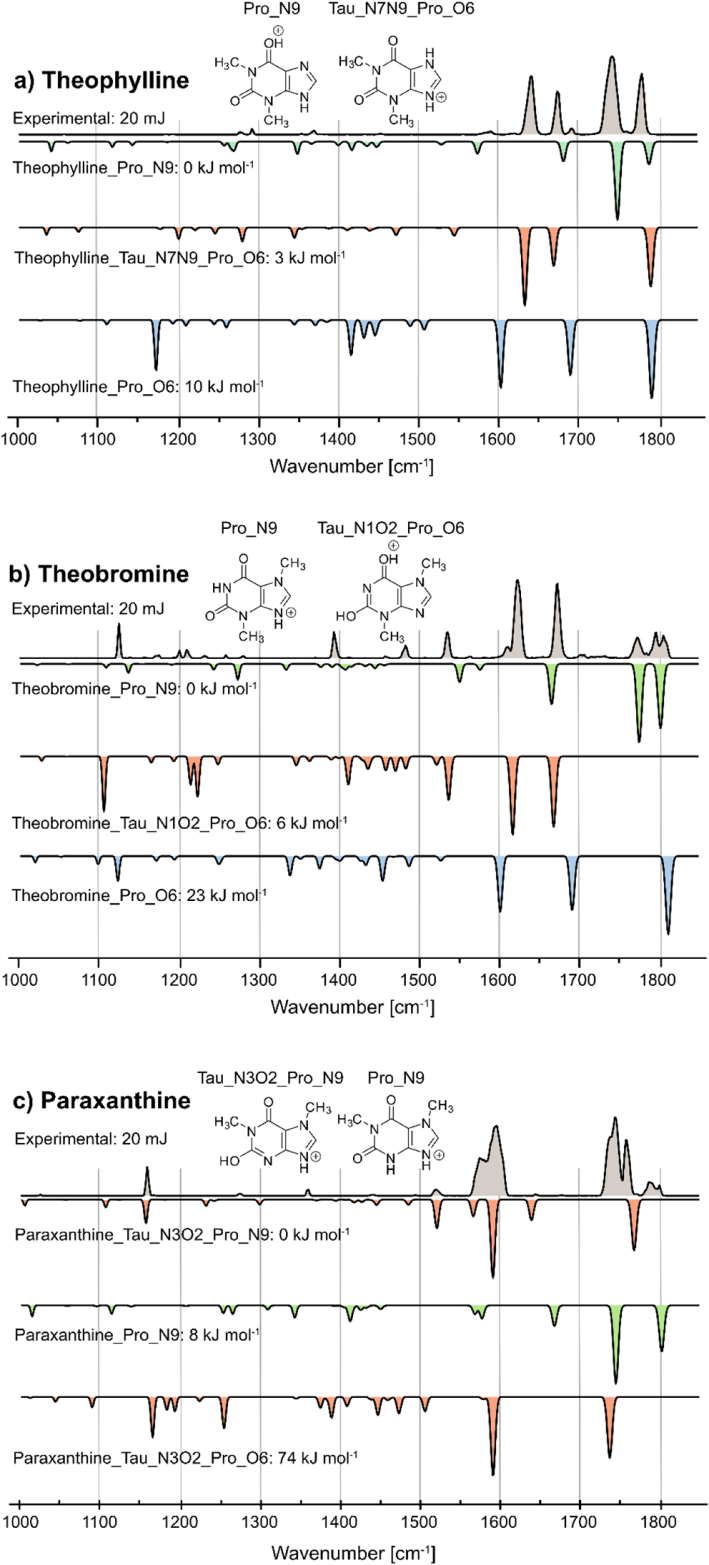
Cryogenic infrared spectra of the protonated cations for: a) theophylline, b) theobromine, and c) paraxanthine. Computed IR spectra are shown as inverted traces for structures with N9-protonation (green), O6-protonation (blue), and different ions with tautomerism (red). For all three species, the two energetically most stable ions were found as the major ions, and their structures are depicted.

For theophylline, the best agreement was found with a mixture of the N9 protomer and the N7N9 tautomeric form that was protonated at the O6 position (Figure 3a). The bands of Pro_N9 can be assigned as those for caffeine (C2=O: 1781 cm^–1^, C6=O: 1744 cm^–1^, C4=N3: 1693 cm^–1^). For the Tau_N7N9_Pro_O6 ion, the C6=O band disappears due to protonation at O6 with two bands appearing at 1675 cm^–1^ and 1642 cm^–1^ as a result of the tautomerism instead. This can be attributed to C=C and C=N vibrations in the bicyclic scaffold. Small amounts of exclusively O6-protonated ions might be present additionally, as indicated by the band at 1621 cm^–1^. The results agree with the relative energetics of the sampled structures and with a previous gas-phase IR spectroscopy study,^38^ however, they are in contrast to cyclic IM-MS work, which only found a single isomer for protonated theophylline.^12^

The second isomer, theobromine, was also previously found to have two isomers with IM-MS,^12^ and the two energetically lowest structures (Pro_N9 and Tau_N1O2_Pro_O6) are indeed the best matches with the experimental data (Figure 3b). Two C=O bands at 1797 cm^–1^ and 1774 cm^–1^ can be clearly assigned to Pro_N9, whereas the two strong peaks at 1674 cm^–1^ and 1624 cm^–1^ are diagnostic of Tau_N1O2_Pro_O6. Notably, the presence of a small third carbonyl band at 1807 cm^–1^, as well as the signals at 1704 cm^–1^ (C=C) and 1611 cm^–1^ (C=N), suggests that Pro_O6 is present as a minor, third isomer.

In the case of paraxanthine, Sepman et al. previously found two isomers,^12^ and the combination of the theoretical IR spectra of the two energetically lowest structures (Tau_N3O2_Pro_N9 and Pro_N9) agrees well with the experimental IR data (Figure 3c). Notably, two minor bands are present at 1645 cm^–1^ and 1677 cm^–1^ in the experimental data (Figure 3c), which were also found theoretically in Tau_N3O2_Pro_N9 and Pro_N9, respectively. There are also a variety of carbonyl bands present in the experimental spectrum, which could suggest additional isomers such as Tau_N3O2_Pro_O6. However, given its high relative energy of 74 kJ mol^–1^ this seems unlikely. In the experimental spectrum, the region between 1550 and 1625 cm^–1^ was remeasured at a macropulse energy of 11 mJ, confirming the presence of two bands (Figure S15).

The group of secondary metabolites—1-methylxanthine, 3-methylxanthine, and 7-methylxanthine—each possess only one methyl group, resulting in more possible combinations of tautomerism and protonation isomers (Figure 1b,c). All three species previously yielded two isomers with IM-MS,^12^ and here we were able to experimentally assign the two main species using cryogenic gas-phase IR spectroscopy.

For 1-methylxanthine (Figure 4a), the two most stable isomers, Tau_N3O2_Pro_N9 and Pro_N9, matched the experimental spectrum well; however, in both the C=O and C=C/C=N regions, various bands are present. This could indicate the presence of other isomers; however, they are considerably higher in energy than the most stable isomer (between 17 and 37 kJ mol^–1^). Hence, only traces would be present, which agrees with the low intensity of several bands.

**Figure 4:**
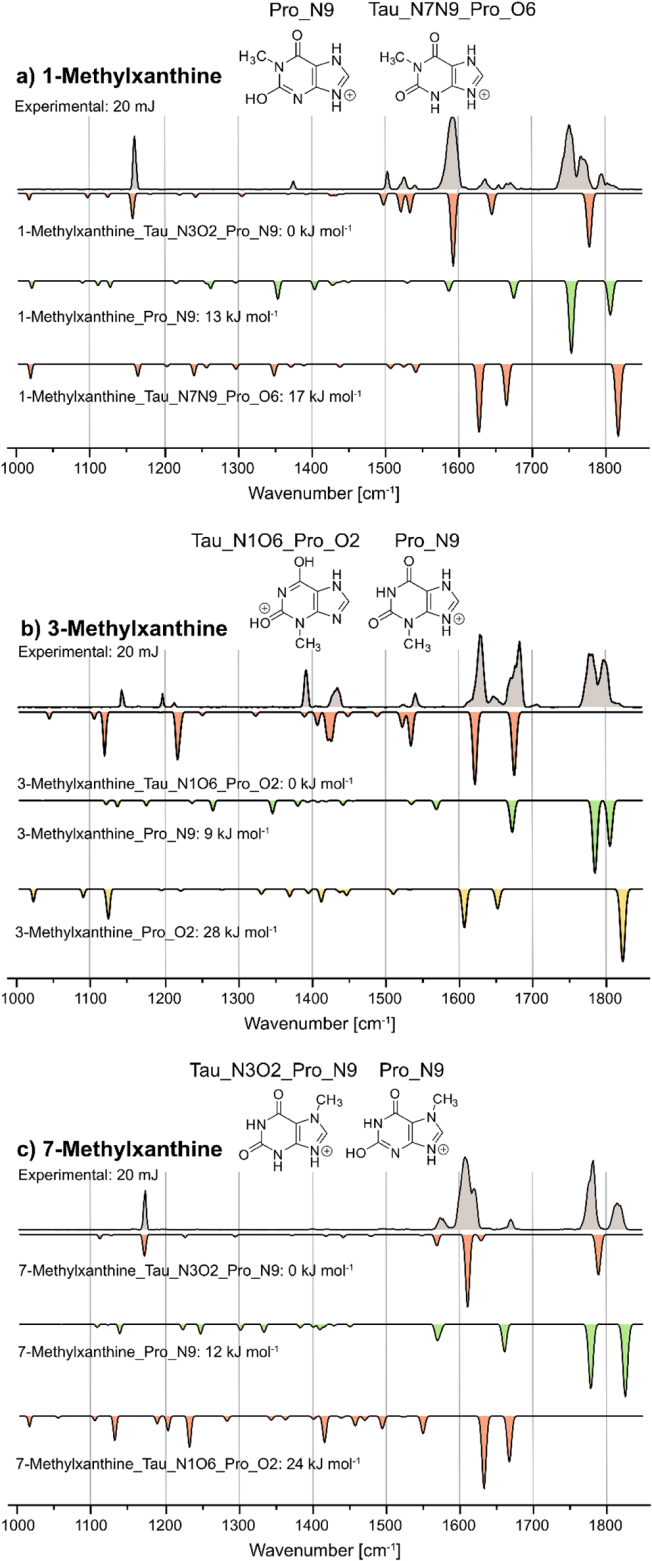
Cryogenic infrared spectra of the protonated cations for: a) 1-methylxanthine, b) 3-methylxanthine, and c) 7-methylxanthine. Computed IR spectra are shown as inverted traces for structures with N9-protonation (green), O2-protonation (yellow), and different ions with tautomerism (red). For all three species, the two energetically most stable ions were found as the major ions, and their structures are depicted.

For 3-methylxanthine, both energetic considerations and the comparison of IR spectra suggest the presence of Tau_N1O6_Pro_O2 and Pro_N9, but a variety of bands were found that could not all be matched with the simulated spectra of these two species (Figure 4b). For clarity, the regions between 1600–1700 cm^–1^ and 1760–1828 cm^–1^ were further resolved at a laser macropulse energy of 11 mJ (Figures S16 and S17). Overall, the data suggest that Pro_O6 and particularly Pro_O2 (due to the band at 1647 cm^–1^) could be present in addition to the two main species; however, an unambiguous identification is not possible.

The theoretical data for 7-methylxanthine yielded Tau_N3O2_Pro_N9 and Pro_N9 as the most stable species, and the combined simulated IR spectra agree well with the experiment (Figure 4c). For clarity, the regions between 1585–1635 cm^–1^ and 1760–1835 cm^–1^ were further resolved at a laser macropulse energy of 11 mJ (Figures S18 and S19), yielding two and three bands in these regions, respectively. No other major signals were found that could not be assigned to either of the two isomers.

## Discussion

In mass spectrometry-based metabolomics, protonated ions are the most likely ions in positive ionization mode, making their structural characterization highly significant. For ions with several basic sites, different isomers can emerge based on varying protonation sites, and these so-called protomers can vary in their structures and properties.^39–41^ Whether the protonation sites in the gas phase resemble those in solution remains debated, and, in particular, the effect of protic and aprotic solvents has been discussed previously.^42–44^ Protic solvents are assumed to act as proton carriers, enabling conversion to the thermodynamically most stable protomer. In contrast, aprotic solvents can kinetically trap energetically less stable protomers.^12,43^ The solvent conditions applied in this study are 4:1 acetonitrile:water with 0.5% formic acid, which is overall highly protic. In all cases, the two energetically lowest isomers were found experimentally as the main peaks, suggesting that the influence of the kinetically trapped protomers is negligible.

The presence of different protomers is often seen as a complication in analytical workflows. However, Kruve and coworkers suggested that differences in protonation preferences could serve as a means of distinguishing regioisomeric caffeine metabolites.^12^ While some isomers showed notable differences in the number of peaks and arrival times of their IM distributions, attributable to protomers and tautomers, some analytes were distinguishable only through high-resolution tandem ion mobility spectrometry (IMS^2^). Here, the authors mobility-isolated and collisionally activated different protomers and tautomers. It was shown that species with protons located at distant heteroatoms (e.g., N9 and O2, Figure 1) cannot interconvert in the absence of solvent, whereas tautomers show intramolecular proton hopping between neighboring heteroatoms in the gas phase. In combination with energy rankings from DFT calculations, they rationalized the IMS^2^ data and distinguished all ions, showcasing the potential of protomers as diagnostic markers for metabolites. While interesting, IMS^2^ has limited practical relevance in omics-based research, and there is therefore a need for complementary analytical tools.

Under the light of developing instrumentation for gas-phase IR spectroscopy,^23–25^ the relevance of this technique for metabolomics and metabolite characterization is growing.^20,21^ This is particularly the case for isomeric ions, such as the caffeine metabolites investigated here, which cannot be differentiated by MS alone and not even directly with high-resolution IM.^12^ Our study shows that protomers of theophylline, theobromine, and paraxanthine, as well as 1-methylxanthine, 3-methylxanthine, and 7-methylxanthine, can readily be distinguished through their vibrational spectral fingerprints (Figure 5). As several classes of metabolites exhibit protomeric and/or tautomeric isomerism, gas-phase IR spectroscopy holds great potential to contribute to their structural characterization and, most importantly, their direct identification.

**Figure 5:**
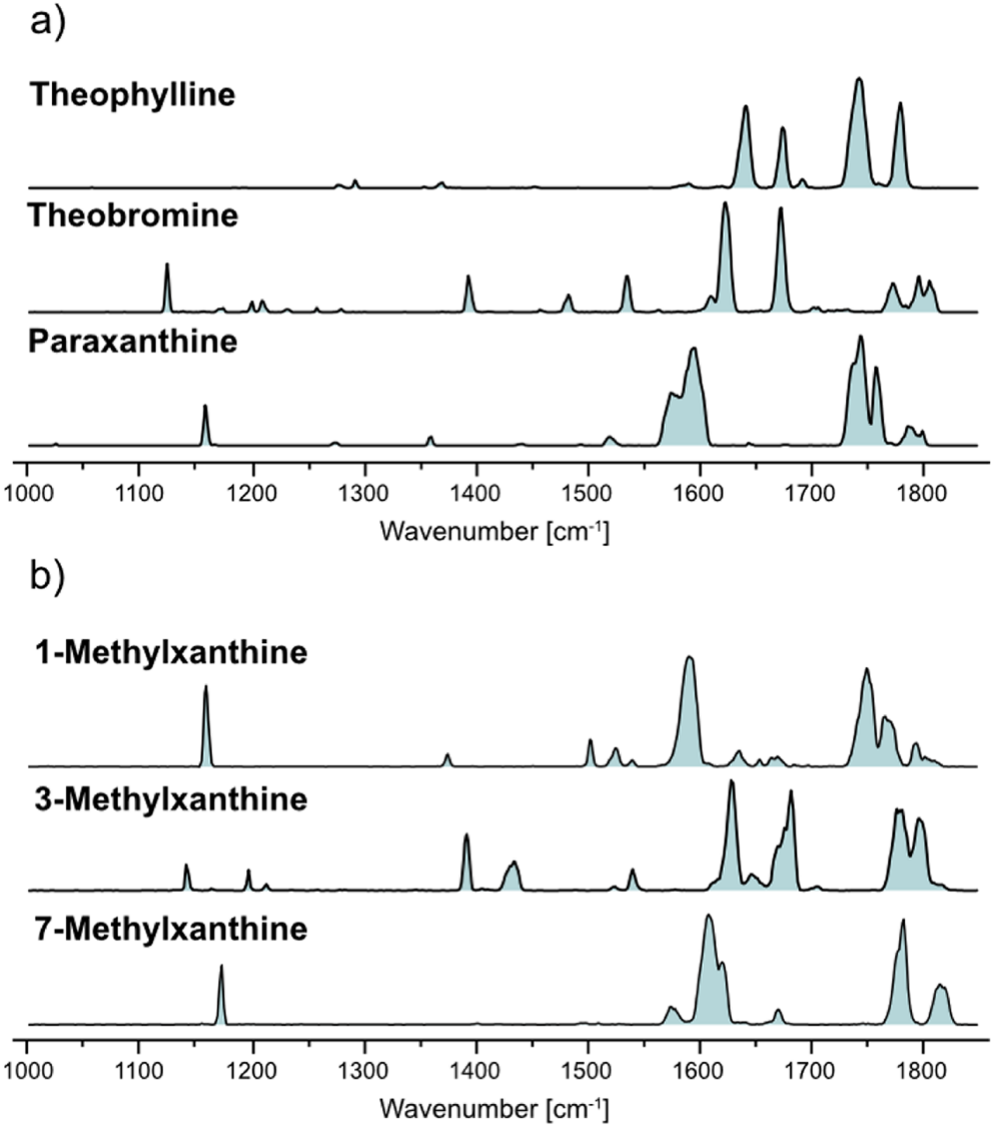
Cryogenic infrared spectra of the protonated cations for: a) primary caffeine metabolites (theophylline, theobromine, and paraxanthine; *m*/*z* = 181), and b) secondary caffeine metabolites (1-methylxanthine, 3-methylxanthine, and 7-methylxanthine; *m*/*z* = 167). All isomers can be readily distinguished through their gas-phase IR fingerprints, which are largely based on the preference for different protomers and/or tautomers.

The presented results may have future implications in metabolomics once they are tested in relevant matrices, particularly for the detection and differentiation of isomeric metabolites in clinical samples. For caffeine metabolites, this can help in considering individual metabolic responses, assessing specific drug interactions (e.g., medication also metabolized via cytochrome P450), as well as monitoring exposure or therapeutic outcomes.^45^ Caffeine pharmacokinetics are generally known for their large interindividual variability, the underlying mechanisms of which are poorly understood.^45^ Caffeine has been suggested to exhibit neuroprotective properties, with potential therapeutic applications in neurodegenerative diseases such as Alzheimer’s disease and Parkinson’s disease.^2^ For a comprehensive understanding of its metabolism and physiological effects, new analytical tools, such as cryogenic gas-phase IR spectroscopy, are warranted to resolve isomeric complexity.

In addition, the data presented here provide valuable insights into the protomeric and tautomeric preferences of methylxanthines. Our results show that, for all studied caffeine metabolites, two main protomers and/or tautomers occur in the gas phase. This generally agrees well with the previous results by Sepman et al. The only exception is theophylline, which, in their study, yielded only one component using high-resolution IM-MS.^12^ The reason for this remains unclear, in particular as the authors used similar solvent conditions to our experiment and reported no solvent effect. One possibility is an overlap in arrival time of both species, even at high resolving power, and this could also explain why, for many of the metabolites, minor isomers were found here in addition to the two species reported by Sepman et al.^12^

The previously suggested isomers and tautomers, based on energetics from DFT calculations, were mostly confirmed by comparing the experimental cryogenic gas-phase IR spectra to those calculated from harmonic frequencies.^12^ One of the two main species is always Pro_N9, showing the high basicity of the N9 atom. For all cases where possible (not caffeine and theophylline), proton hopping through amine–imidic acid tautomerism was found to occur at O2, with N3O2 tautomerism being preferred over N1O2 tautomerism, as the example of 7-methylxanthine suggests. While protonation occurs mostly at the N9 site, for those molecules without a methyl group at N1, protonation can occur at the O6 site in combination with N1O2 tautomerism (i.e., same as the O2 protonation with N1–O6 tautomerism). Lastly, N7N9 imine–imine tautomerism was found only in theophylline, where no other type of tautomerism is possible.

## Conclusions

Taken together, we demonstrate the potential of cryogenic gas-phase IR spectroscopy to unambiguously distinguish isomeric caffeine metabolites, which, until now, has not been directly possible with other gas-phase techniques such as IM-MS. Supported by DFT calculations, we elucidate the structures of the occurring protomers and tautomers for each analyte, revealing the preference for protonation at N9 and the occurrence of N3O2, N1O2, and N7N9 tautomerism, where possible. While the variety of gas-phase protomers and tautomers can complicate structural characterization, they can also yield unique vibrational fingerprints that enable their identification with cryogenic gas-phase IR spectroscopy. We believe that this technique will be a robust addition to the analytical toolbox of omics research, in particular, for protomers and tautomers.

## Supplementary Material



## Data Availability

The supplementary dataset is available on Figshare (DOI: 10.6084/m9.figshare.29959565) and contains the raw data for mass spectrometry and gas-phase infrared spectroscopy experiments as well as density functional theory calculations.

## Notes

Contains mass spectra; energetics and structures of densitiy functional theory computed candidates; and gas-phase IR spectra of certain regions at lower macropulse energies (PDF)
