# Supplementary material for: Distinguishing Isomeric Caffeine Metabolites through Protomers and Tautomers Using Cryogenic Gas-Phase Infrared Spectroscopy
Source: Anal Chem. 2025 Sep 25;97(39):21740–7. doi: 10.1021/acs.analchem.5c05164 (PMC12509192; doi:10.1021/acs.analchem.5c05164)
Supplement: Supplementary file 1 [file ac5c05164_si_001.pdf]

Supporting Information

Distinguishing Isomeric Caffeine Metabolites through Protomers and Tautomers using Cryogenic Gas-Phase Infrared Spectroscopy

Niklas Geue[1,2,*], Gurpur Rakesh D. Prabhu[1,2], Eleonora Renzi[1,2], Caitlin Walton-Doyle[1,2], Gerard Meijer[2], Gert von Helden[2] and Kevin Pagel[1,2,*]

[1]Institute of Chemistry and Biochemistry, Freie Universität Berlin, Altensteinstraße 23a, 14195 Berlin, Germany. [2]Department of Molecular Physics, Fritz-Haber-Institut der Max-Planck-Gesellschaft, Faradayweg 4–6, 14195 Berlin, Germany.

[*]Corresponding Authors: niklas.geue@fu-berlin.de, kevin.pagel@fu-berlin.de

Table of Content

Figure S1: Mass spectrum of caffeine 3

Figure S2: Mass spectrum of theophylline 4

Figure S3: Mass spectrum of theobromine 5

Figure S4: Mass spectrum of paraxanthine 6

Figure S5: Mass spectrum of 1-methylxanthine 7

Figure S6: Mass spectrum of 3-methylxanthine 8

Figure S7: Mass spectrum of 7-methylxanthine 9

Table S1: Energetics of protonated caffeine structures 10

Table S2: Energetics of protonated theophylline structures 11

Table S3: Energetics of protonated theobromine structures 12

Table S4: Energetics of protonated paraxanthine structures 13

Table S5: Energetics of protonated 1-methylxanthine structures 14

Table S6: Energetics of protonated 3-methylxanthine structures 15

Table S7: Energetics of protonated 7-methylxanthine structures 16

Figure S8: DFT optimised structures of caffeine with protomers and tautomers. 17

Figure S9: DFT optimised structures of theophylline with protomers and tautomers. 18

Figure S10: DFT optimised structures of theobromine with protomers and tautomers. 19

Figure S11: DFT optimised structures of paraxanthine with protomers and tautomers. 20

Figure S12: DFT optimised structures of 1-methylxanthine with protomers and tautomers. 21

Figure S13: DFT optimised structures of 3-methylxanthine with protomers and tautomers. 22

Figure S14: DFT optimised structures of 7-methylxanthine with protomers and tautomers. 23

Figure S15: Cryogenic infrared spectrum of the protonated cation of paraxanthine at 11 mJ in the region from 1550 to 1625 cm^{-1}. 24

Figure S16: Cryogenic infrared spectrum of the protonated cation of 3-methylxanthine at 11 mJ in the region from 1600 to 1700 cm^{-1}. 25

Figure S17: Cryogenic infrared spectrum of the protonated cation of 3-methylxanthine at 11 mJ in the region from 1760 to 1828 cm^{-1}. 26

Figure S18: Cryogenic infrared spectrum of the protonated cation of 7-methylxanthine at 11 mJ in the region from 1585 to 1635 cm^{-1}. 27

Figure S19: Cryogenic infrared spectrum of the protonated cation of 7-methylxanthine at 11 mJ in the region from 1760 to 1835 cm^{-1}. 28

References .... Error! Bookmark not defined.

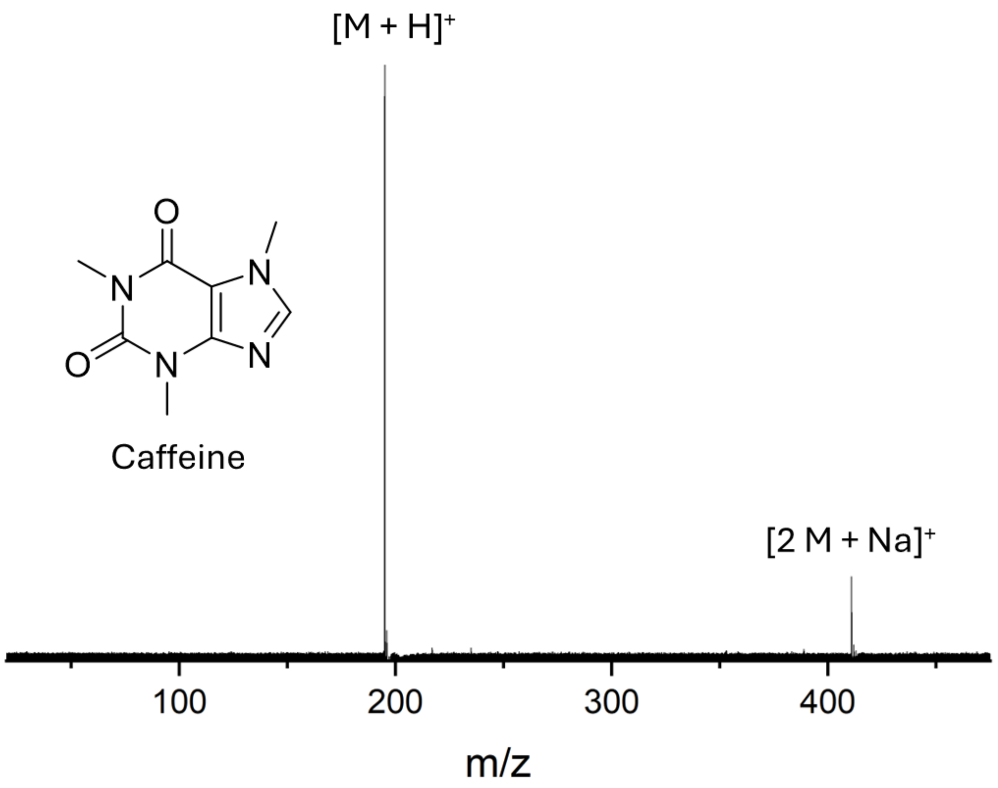

Figure S1: Mass spectrum of caffeine in 4:1 acetonitrile/water (v/v) with 0.5% formic acid (final concentration: 200 µM). Apart from the protonated monomer, the sodiated dimer is clearly visible.

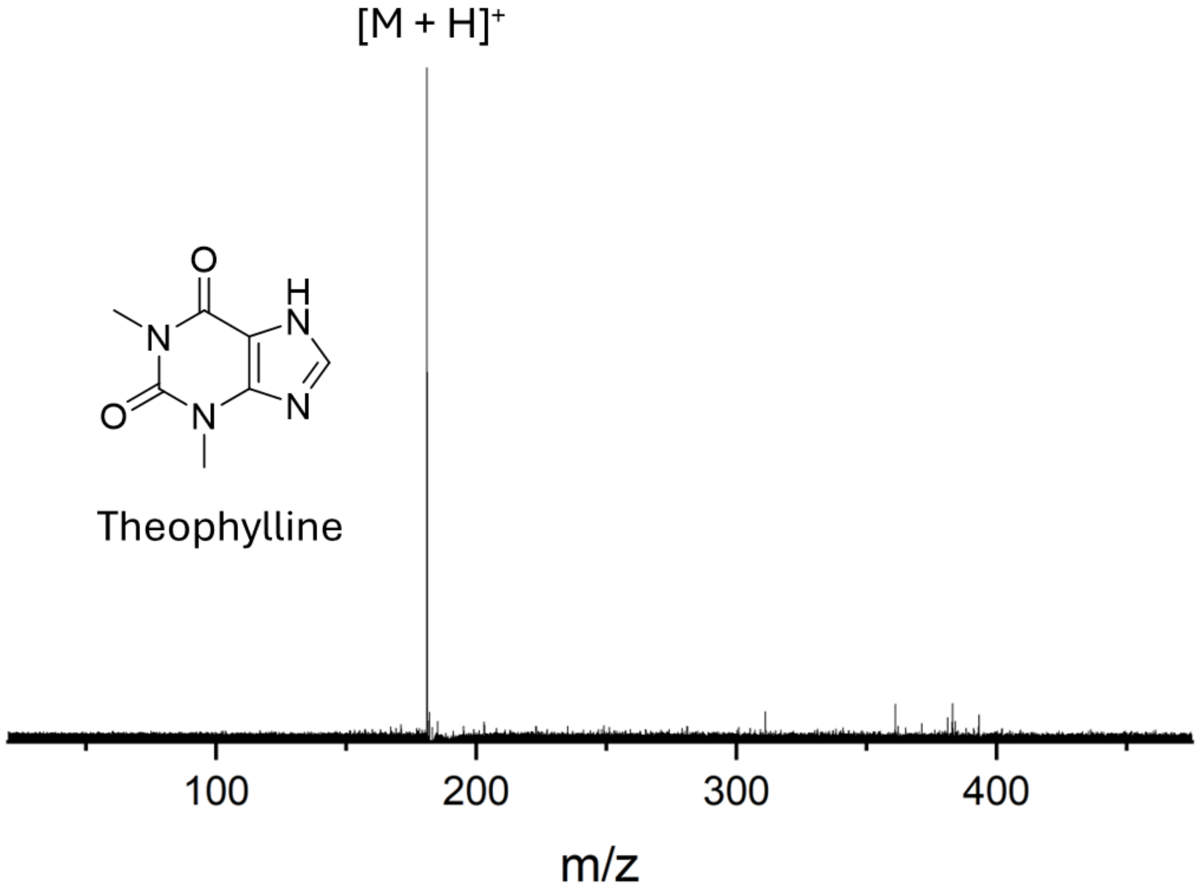

Figure S2: Mass spectrum of theophylline in 4:1 acetonitrile/water (v/v) with 0.5% formic acid (final concentration: 200 µM). The protonated monomer was found as the main peak.

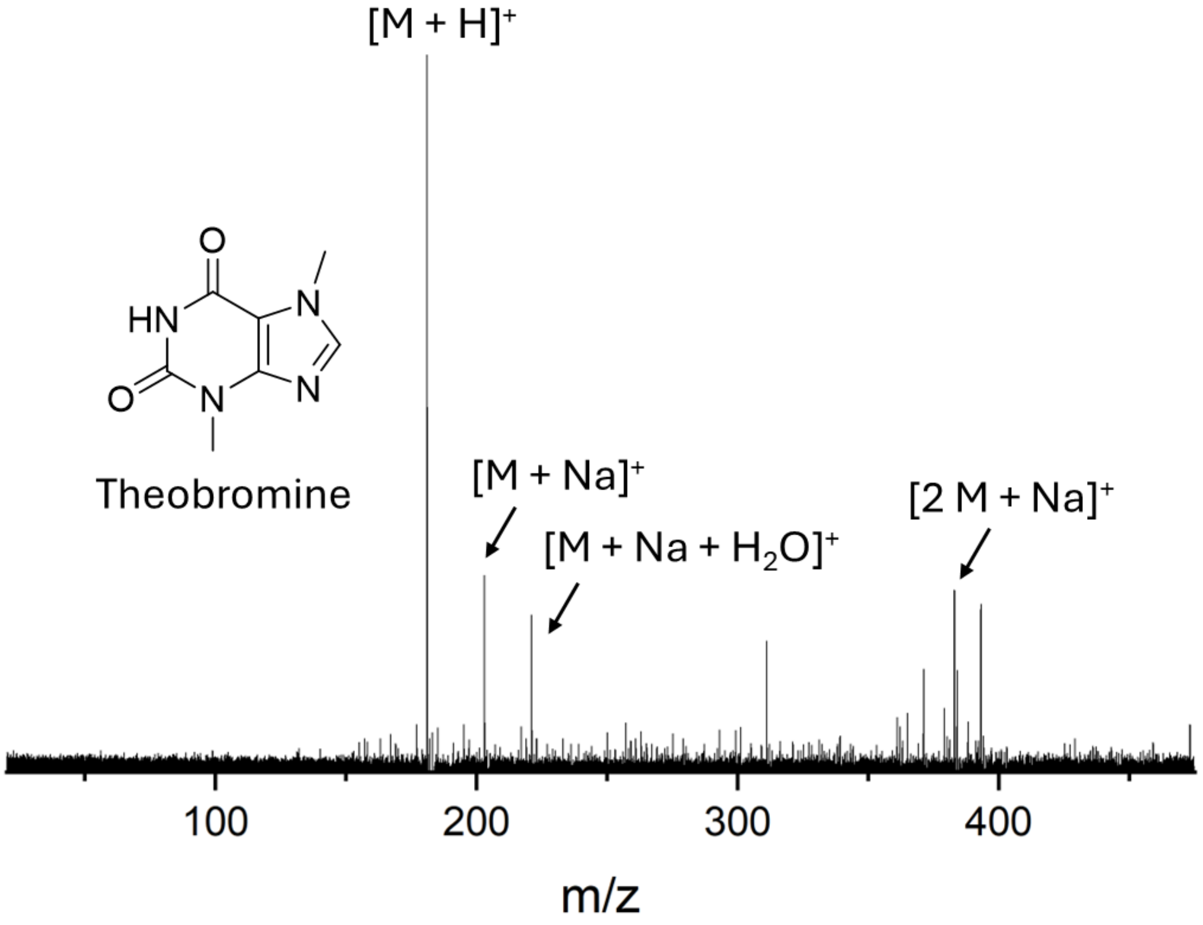

Figure S3: Mass spectrum of theobromine in 4:1 acetonitrile/water (v/v) with 0.5% formic acid (final concentration: 200 µM). The protonated monomer was found as the main peak, with sodiated monomer and dimer species also being present.

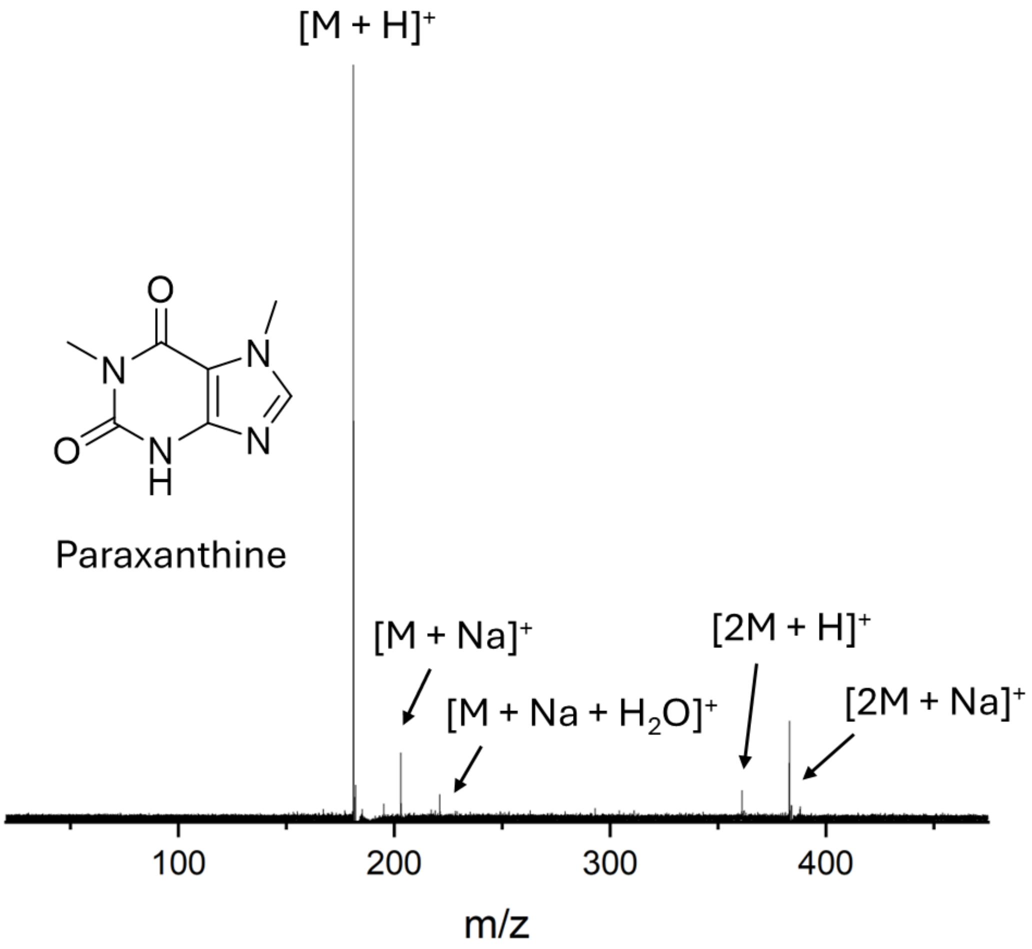

Figure S4: Mass spectrum of paraxanthine in 4:1 acetonitrile/water (v/v) with 0.5% formic acid (final concentration: 200 µM). The protonated monomer was found as the main peak, with sodiated monomer and protonated/sodiated dimer species also being present.

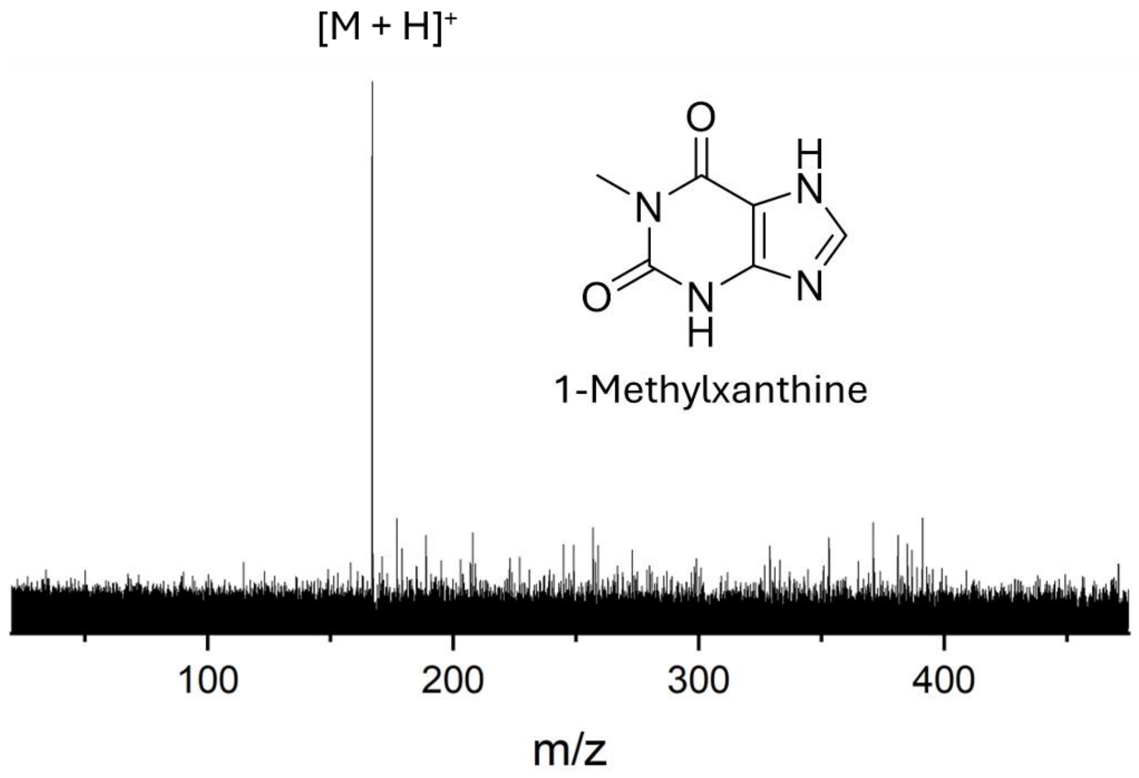

Figure S5: Mass spectrum of 1-methylxanthine in 4:1 acetonitrile/water (v/v) with 0.5% formic acid (final concentration: 1 mM). The protonated monomer was found as the main peak.

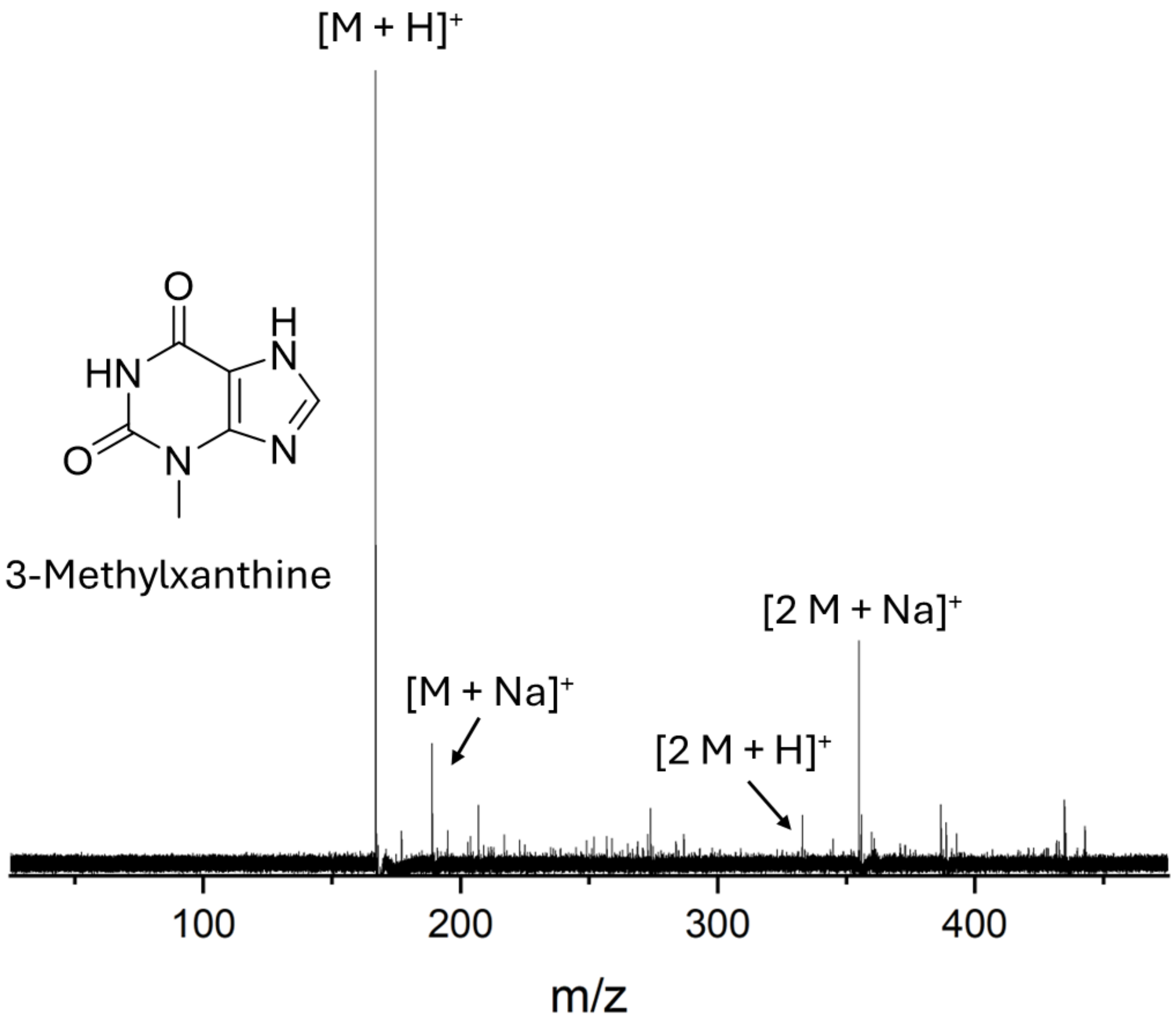

Figure S6: Mass spectrum of 3-methylxanthine in 4:1 acetonitrile/water (v/v) with 0.5% formic acid (final concentration: 1 mM). The protonated monomer was found as the main peak, with sodiated monomer and protonated/sodiated dimer species also being present.

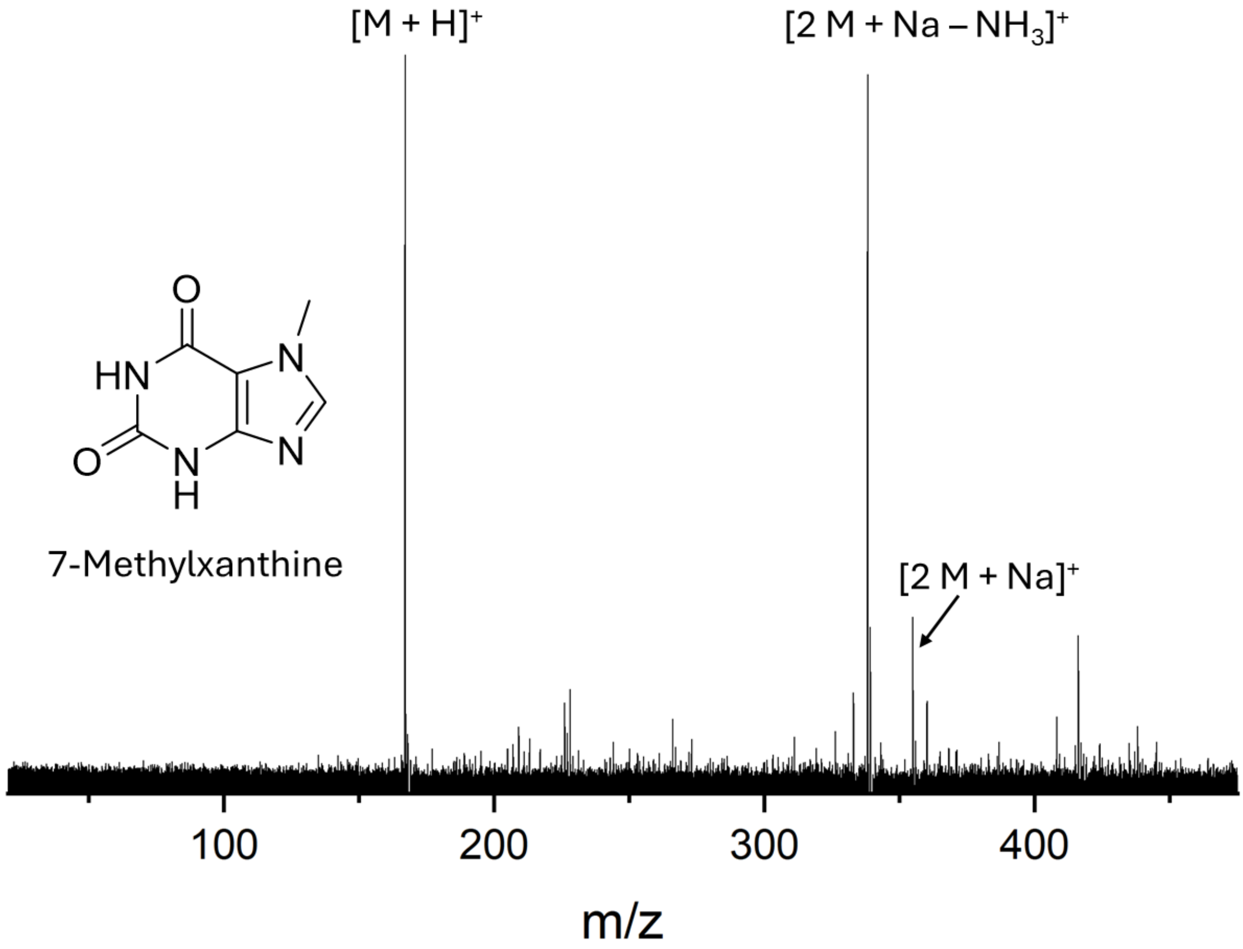

Figure S7: Mass spectrum of 7-methylxanthine in 4:1 acetonitrile/water (v/v) with 0.5% formic acid (final concentration: 1 mM). The protonated monomer was found as the main peak, with the sodiated dimer also being present, as well as a fragment from the dimer likely corresponding to the loss of ammonia.

Table S1: Energetics of protonated caffeine structures optimized at the PBE0+D3/6-311+G(d,p) level of theory. Energies (ΔE, including zero-point-vibrational energy) and free energies (ΔF) at 90 K are assigned to each structure. The infrared spectra of the structures labelled with an asterisk are represented in the manuscript.

ID	ΔE(PBE0 + D3) [kJ mol^{-1}]	ΔF(PBE0 + D3) [kJ mol^{-1}]
Caffeine_Pro_N9 (*)	0.00	0.00
Caffeine_Pro_O6 (*)	17.85	15.79
Caffeine_Pro_O2 (*)	29.61	28.25

Table S2: Energetics of protonated theophylline structures optimized at the PBE0+D3/6-311+G(d,p) level of theory. Energies (ΔE, including zero-point-vibrational energy) and free energies (ΔF) at 90 K are assigned to each structure. The infrared spectra of the structures labelled with an asterisk are represented in the manuscript.

**ID**	**ΔE(PBE0 + D3) [kJ mol^{-1}]**	**ΔF(PBE0 + D3) [kJ mol^{-1}]**
Theophylline_Pro_N9 (*)	0.00	0.00
Theophylline_Tau_N7N9_Pro_O6 (*)	3.98	3.54
Theophylline_Pro_O6 (*)	10.33	10.45
Theophylline_Pro_O2	24.52	25.63
Theophylline_Tau_N7N9_Pro_O2	74.62	75.57

Table S3: Energetics of protonated theobromine structures optimized at the PBE0+D3/6-311+G(d,p) level of theory. Energies (ΔE, including zero-point-vibrational energy) and free energies (ΔF) at 90 K are assigned to each structure. The infrared spectra of the structures labelled with an asterisk are represented in the manuscript.

ID	ΔE(PBE0 + D3) [kJ mol^{-1}]	ΔF(PBE0 + D3) [kJ mol^{-1}]
Theobromine_Pro_N9 (*)	0.00	0.00
Theobromine_Tau_N1O2_Pro_O6 (*)	4.14	5.91
Theobromine_Pro_O6 (*)	24.37	23.07
Theobromine_Pro_O2	29.75	28.15
Theobromine_Tau_N1O2_Pro_N9	61.83	61.95
Theobromine_Tau_N1O6_Pro_N9	114.31	112.98

Table S4: Energetics of protonated paraxanthine structures optimized at the PBE0+D3/6-311+G(d,p) level of theory. Energies (ΔE, including zero-point-vibrational energy) and free energies (ΔF) at 90 K are assigned to each structure. The infrared spectra of the structures labelled with an asterisk are represented in the manuscript.

**ID**	**ΔE(PBE0 + D3) [kJ mol^{-1}]**	**ΔF(PBE0 + D3) [kJ mol^{-1}]**
Paraxanthine_Tau_N3O2_Pro_N9 (*)	0.00	0.00
Paraxanthine_Pro_N9 (*)	11.14	8.39
Paraxanthine_Pro_O6	35.39	34.14
Paraxanthine_Pro_O2	46.04	45.43
Paraxanthine_Tau_N3O2_Pro_O6 (*)	75.73	74.25

Table S5: Energetics of protonated 1-methylxanthine structures optimized at the PBE0+D3/6-311+G(d,p) level of theory. Energies (ΔE, including zero-point-vibrational energy) and free energies (ΔF) at 90 K are assigned to each structure. The infrared spectra of the structures labelled with an asterisk are represented in the manuscript.

ID	ΔE(PBE0 + D3) [kJ mol^{-1}]	ΔF(PBE0 + D3) [kJ mol^{-1}]
1-Methylxanthine_Tau_N3O2_Pro_N9 (*)	0.00	0.00
1-Methylxanthine_Pro_N9 (*)	13.31	12.71
1-Methylxanthine_Tau_N7N9_Pro_O6 (*)	18.52	16.67
1-Methylxanthine_Tau_N7N9_N3O2_Pro_O6	20.59	20.73
1-Methylxanthine_Pro_O6	26.07	24.62
1-Methylxanthine_ Pro_O2	37.98	37.45
1-Methylxanthine_Tau_N3O2_Pro_O6	64.65	63.05
1-Methylxanthine_Tau_N7N9_Pro_O2	83.28	82.57

Table S6: Energetics of protonated 3-methylxanthine structures optimized at the PBE0+D3/6-311+G(d,p) level of theory. Energies (ΔE, including zero-point-vibrational energy) and free energies (ΔF) at 90 K are assigned to each structure. The infrared spectra of the structures labelled with an asterisk are represented in the manuscript.

ID	ΔE(PBE0 + D3) [kJ mol^{-1}]	ΔF(PBE0 + D3) [kJ mol^{-1}]
3-Methylxanthine_Tau_N1O6_Pro_O2 (*)	0.00	0.00
3-Methylxanthine_Pro_N9 (*)	9.74	9.16
3-Methylxanthine_Pro_O6	22.57	20.60
3-Methylxanthine_ Pro_O2 (*)	29.46	27.51
3-Methylxanthine_Tau_N7N9_N1O6_Pro_O2	34.59	33.06
3-Methylxanthine_Tau_N7N9_Pro_O6	40.64	39.05
3-Methylxanthine_Tau_N1O6_Pro_N9	66.36	64.61
3-Methylxanthine_Tau_N7N9_Pro_O2	83.59	82.75
3-Methylxanthine_Tau_N1O2_Pro_N9	124.23	122.57

Table S7: Energetics of protonated 7-methylxanthine structures optimized at the PBE0+D3/6-311+G(d,p) level of theory. Energies (ΔE, including zero-point-vibrational energy) and free energies (ΔF) at 90 K are assigned to each structure. The infrared spectra of the structures labelled with an asterisk are represented in the manuscript.

ID	ΔE(PBE0 + D3) [kJ mol^{-1}]	ΔF(PBE0 + D3) [kJ mol^{-1}]
7-Methylxanthine_Tau_N3O2_Pro_9N (*)	0.00	0.00
7-Methylxanthine_Pro_N9 (*)	12.26	11.77
7-Methylxanthine_Tau_N1O6_Pro_O2 (*)	22.75	24.08
7-Methylxanthine_Tau_N1O6_N3O2_Pro_N9	34.82	36.25
7-Methylxanthine_Pro_O6	43.84	43.50
7-Methylxanthine_Pro_O2	49.28	48.66
7-Methylxanthine_Tau_N3O2_Pro_O6	78.72	78.42
7-Methylxanthine_Tau_N1O6_ Pro_N9	79.17	79.95
7-Methylxanthine_Tau_N1O2_ Pro_N9	121.00	120.50

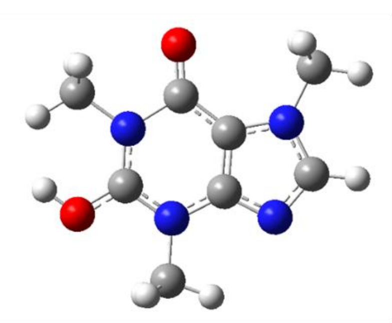

Caffeine_Pro_O2

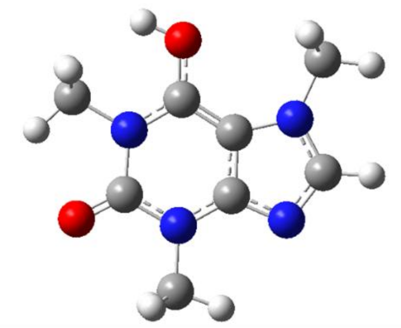

Caffeine_Pro_O6

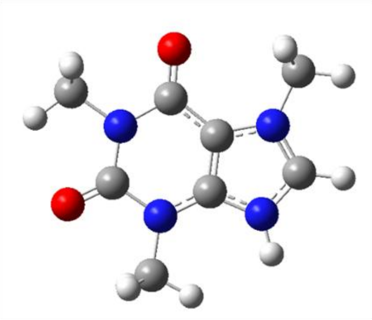

Caffeine_Pro_N9

Figure S8: DFT optimised structures of caffeine with protomers and tautomers.

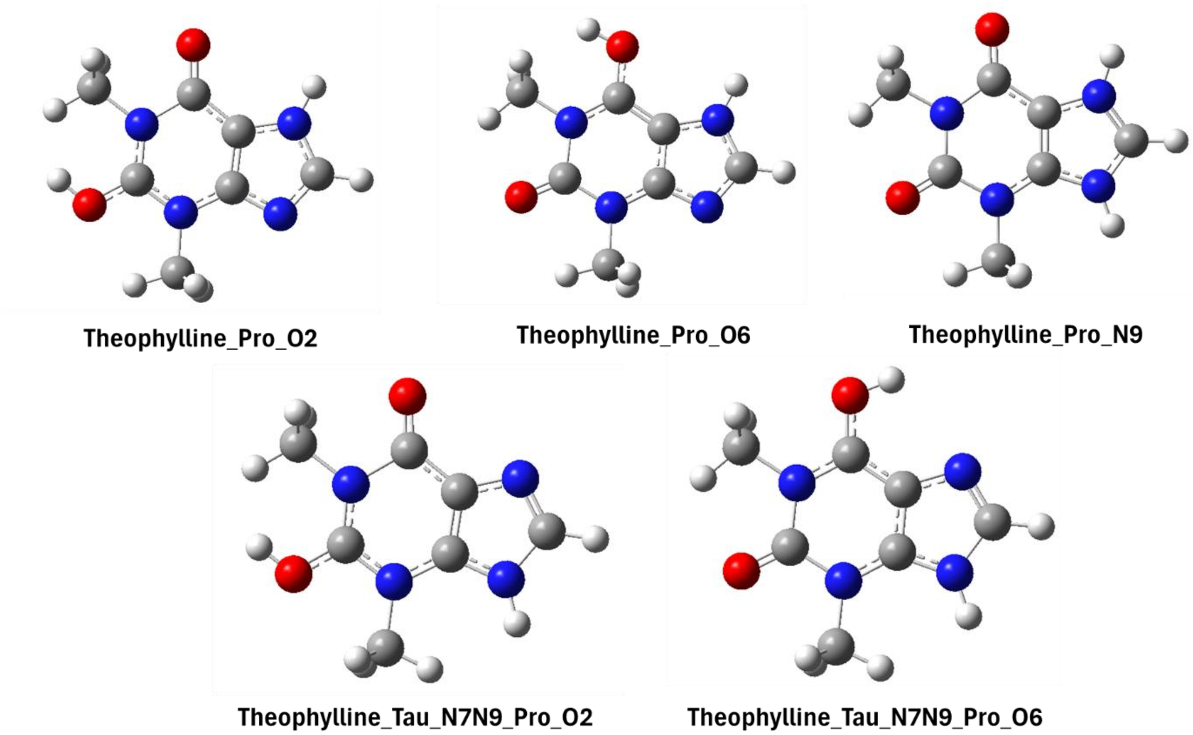

Figure S9: DFT optimised structures of theophylline with protomers and tautomers.

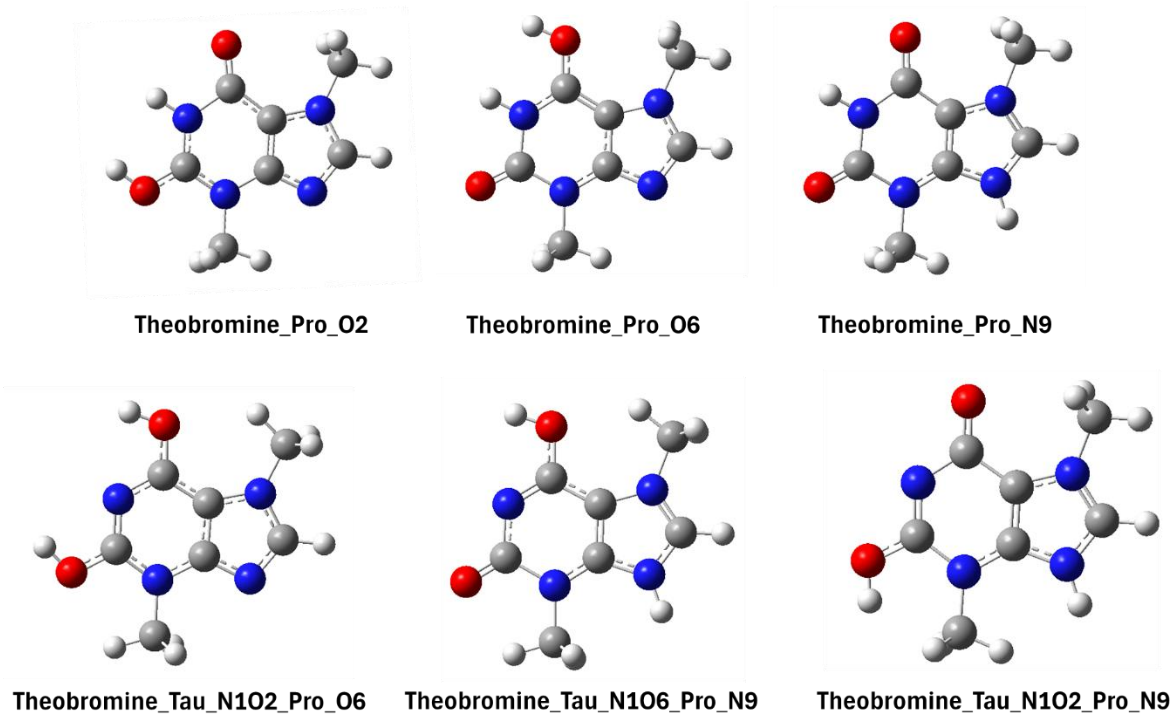

Figure S10: DFT optimised structures of theobromine with protomers and tautomers.

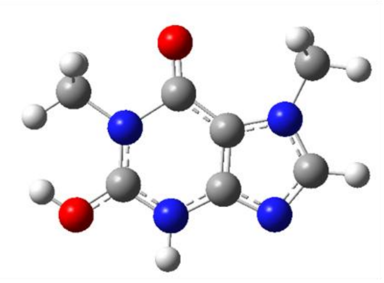

Paraxanthine_Pro_O2

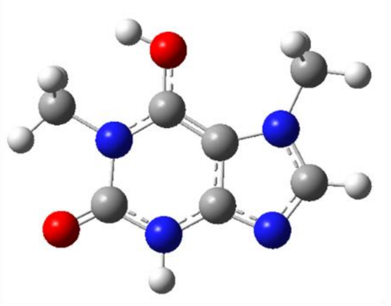

Paraxanthine_Pro_O6

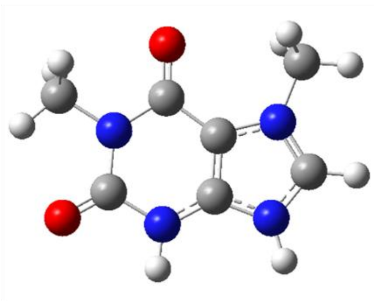

Paraxanthine_Pro_9N

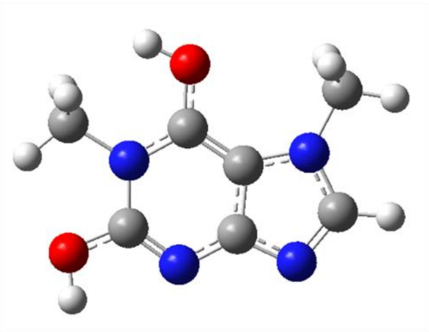

Paraxanthine_Tau_N3O2_Pro_O6

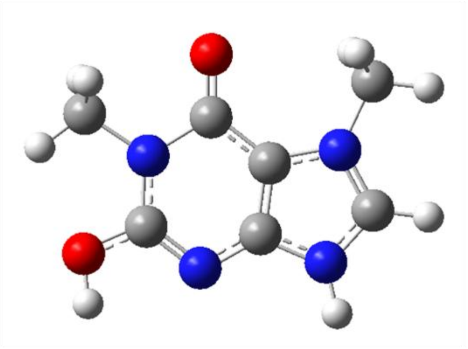

Paraxanthine_Tau_N3O2_Pro_N9

Figure S11: DFT optimised structures of paraxanthine with protomers and tautomers.

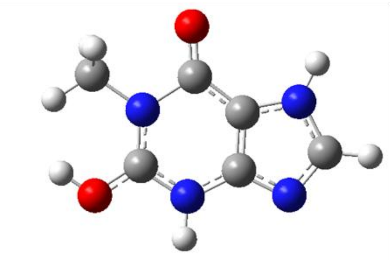

1-Methylxanthine_Pro_O2

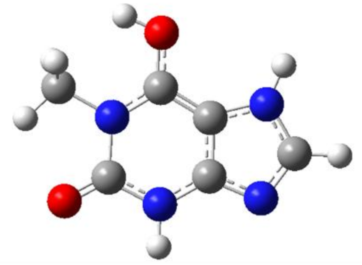

1-Methylxanthine _Pro_O6

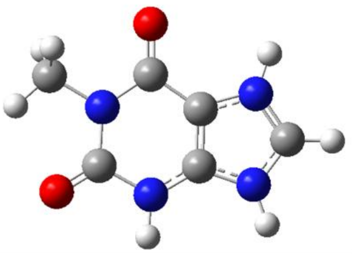

1-Methylxanthine _Pro_9N

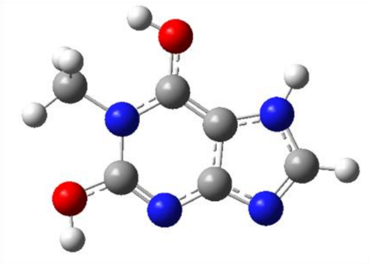

1-Methylxanthine_Tau_N3O2_Pro_O6

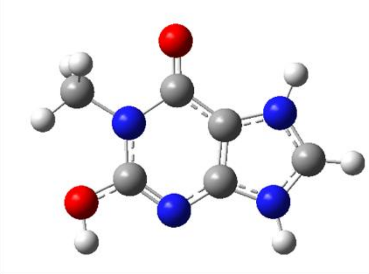

1-Methylxanthine_Tau_N3O2_Pro_N9

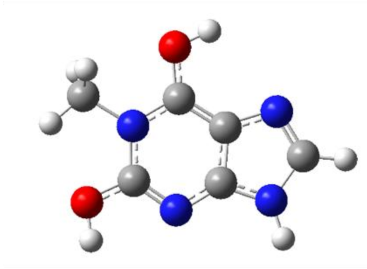

1-Methylxanthine_Tau_N7N9_N3O2_Pro_O6

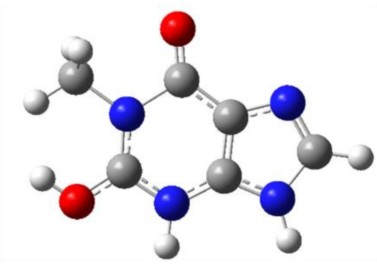

1-Methylxanthine_Tau_N7N9_Pro_O2

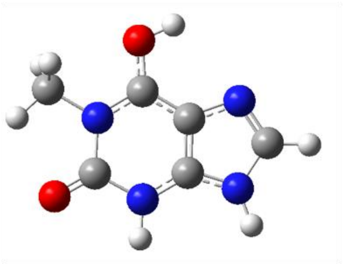

1-Methylxanthine_Tau_N7N9_Pro_O6

Figure S12: DFT optimised structures of 1-methylxanthine with protomers and tautomers.

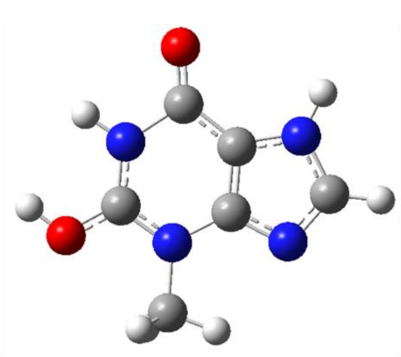

3-Methylxanthine_Pro_O2

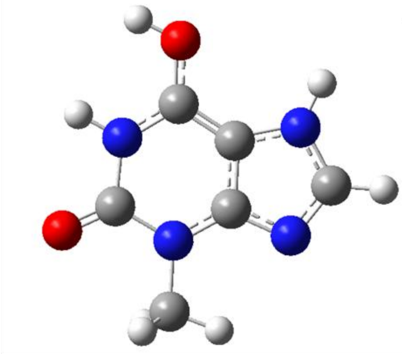

3-Methylxanthine _Pro_O6

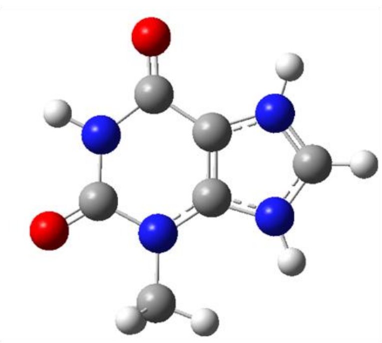

3-Methylxanthine _Pro_9N

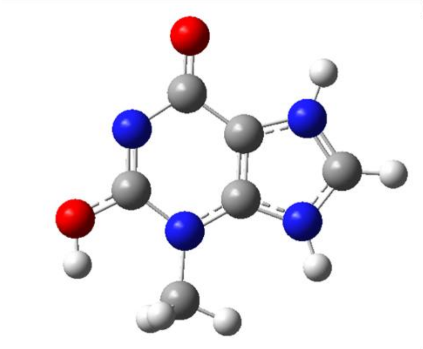

3-Methylxanthine_Tau_N1O2_Pro_N9

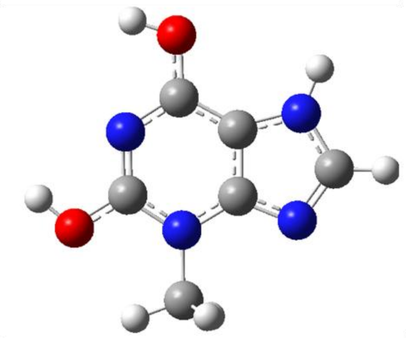

3-Methylxanthine_Tau_N1O6_Pro_O2

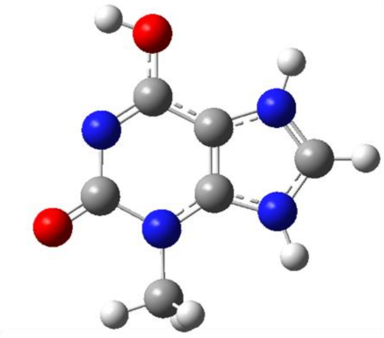

3-Methylxanthine_Tau_N1O6_Pro_N9

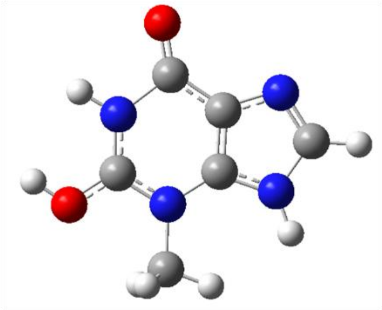

3-Methylxanthine_Tau_N7N9_Pro_O2

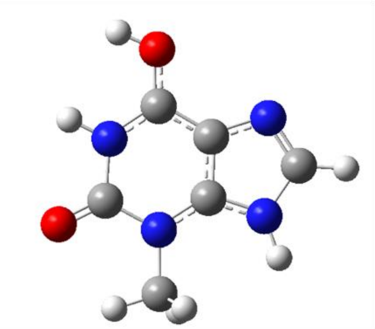

3-Methylxanthine_Tau_N7N9_Pro_O6

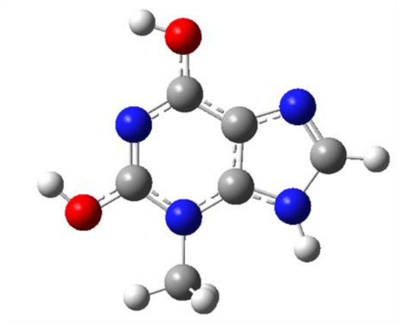

3-Methylxanthine_Tau_N7N9_N1O6_Pro_O2

Figure S13: DFT optimised structures of 3-methylxanthine with protomers and tautomers.

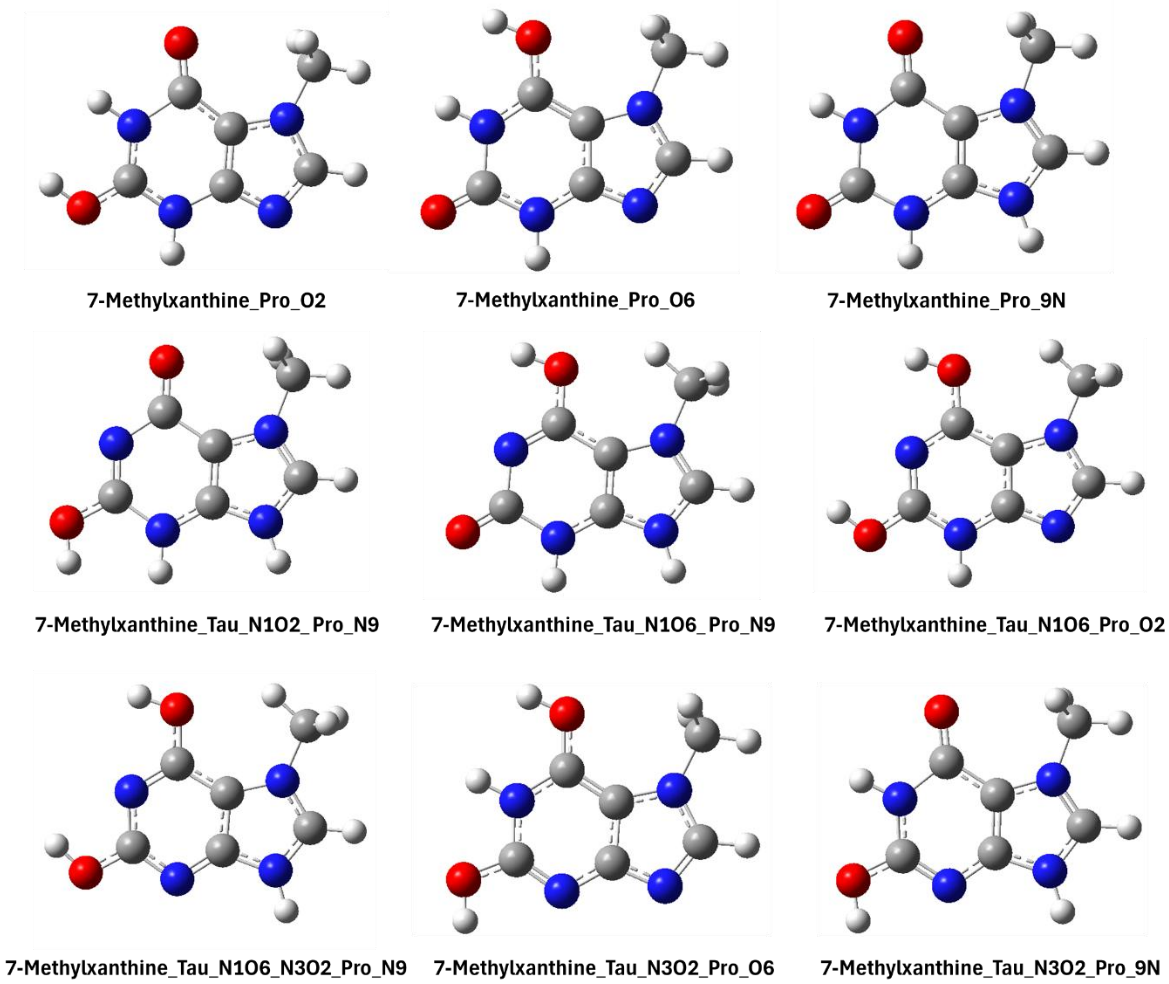

Figure S14: DFT optimised structures of 7-methylxanthine with protomers and tautomers.

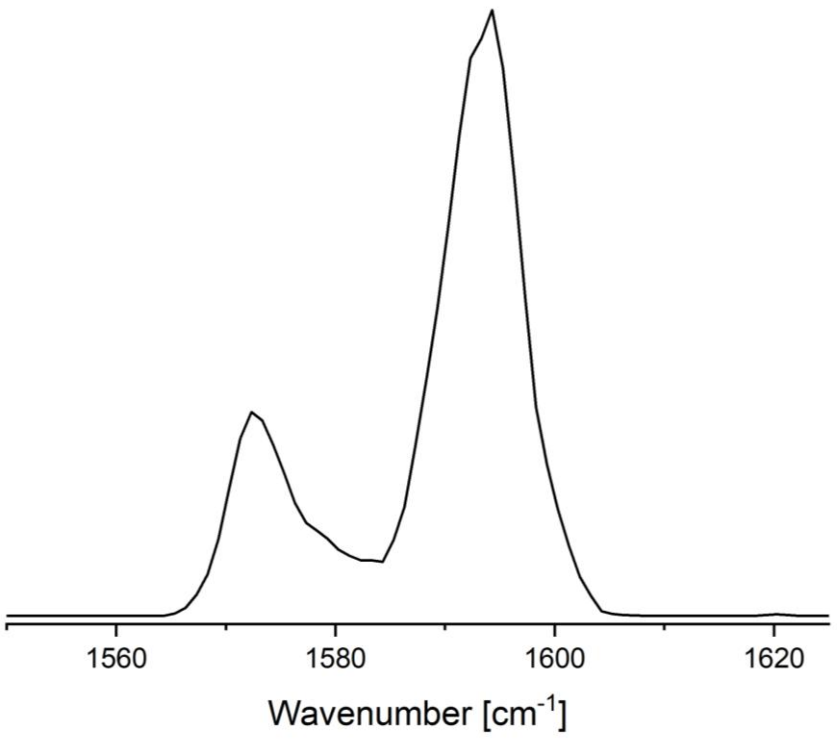

Figure S15: Cryogenic infrared spectrum of the protonated cation of paraxanthine at 11 mJ in the region from 1550 to 1625 cm^{-1}.

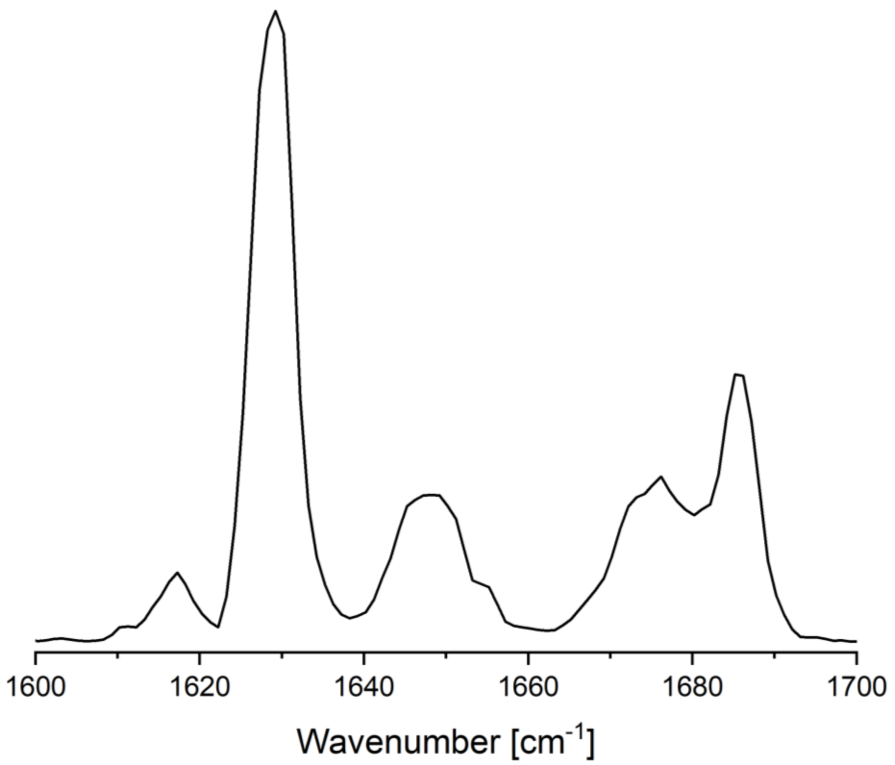

Figure S16: Cryogenic infrared spectrum of the protonated cation of 3-methylxanthine at 11 mJ in the region from 1600 to 1700 cm^{-1}.

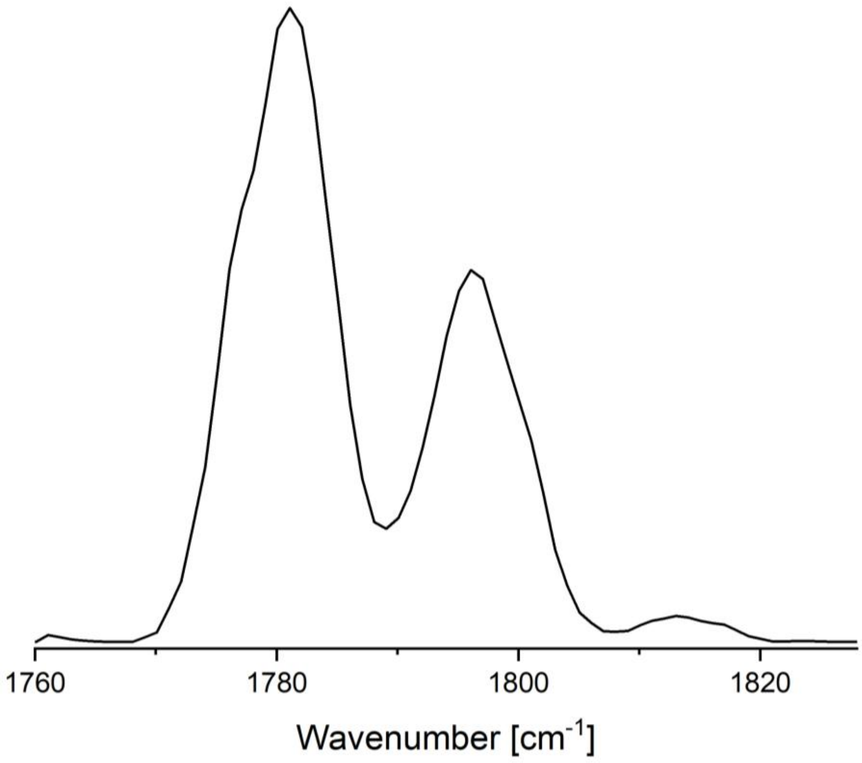

Figure S17: Cryogenic infrared spectrum of the protonated cation of 3-methylxanthine at 11 mJ in the region from 1760 to 1828 cm^{-1}.

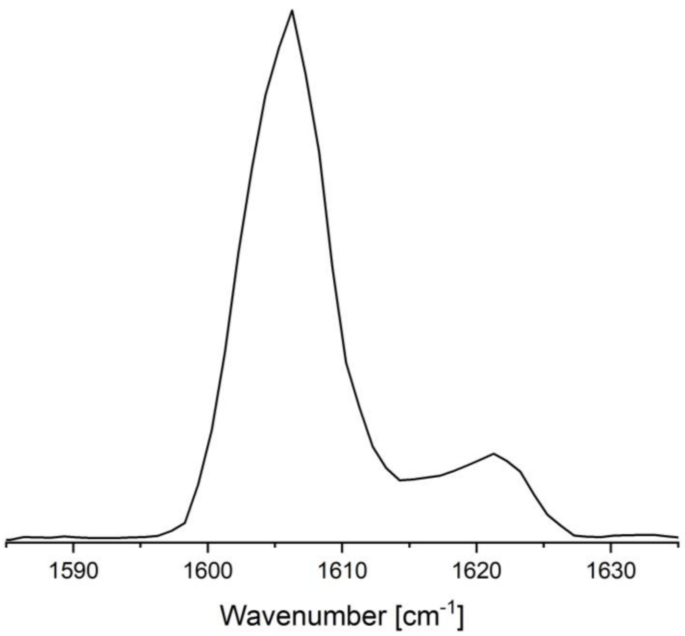

Figure S18: Cryogenic infrared spectrum of the protonated cation of 7-methylxanthine at 11 mJ in the region from 1585 to 1635 cm^{-1}.

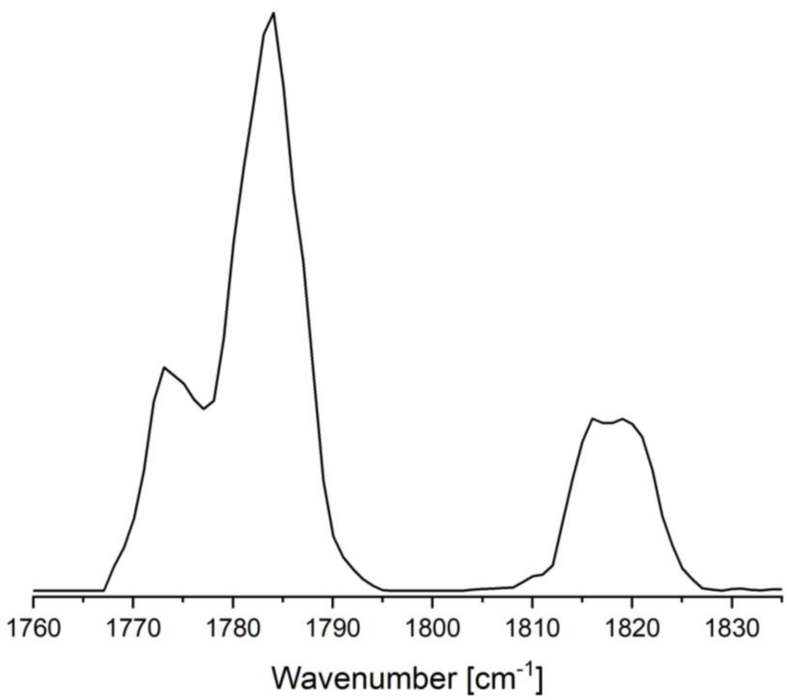

Figure S19: Cryogenic infrared spectrum of the protonated cation of 7-methylxanthine at 11 mJ in the region from 1760 to 1835 cm^{-1}.
